# Plasmids Carrying Antimicrobial Resistance Genes in Gram-Negative Bacteria

**DOI:** 10.3390/microorganisms10081678

**Published:** 2022-08-20

**Authors:** Nadezhda K. Fursova, Angelina A. Kislichkina, Olga E. Khokhlova

**Affiliations:** 1 Department of Molecular Microbiology, State Research Center for Applied Microbiology and Biotechnology, Territory “Kvartal A”, 142279 Obolensk, Russia; 2 Department of Culture Collection, State Research Center for Applied Microbiology and Biotechnology, Territory “Kvartal A”, 142279 Obolensk, Russia

Gram-negative bacteria are prevalent pathogens associated with hospital-acquired infections (HAI) that are a major challenge for patient safety, especially in intensive care units [1]. These infections are extremely important worldwide because they aggravate the course of the underlying diseases, prolong the therapy of patients, and increase the treatment cost and mortality. One of the difficulties of maintenance of patients with HAI is the resistance of the infectious agents to traditional pharmacotherapy. Currently, high rate of emergence and spread of antimicrobial resistance among clinical bacterial strains represents the global health care crisis. It was estimated that drug-resistant infections contribute to nearly 5 million deaths every year [2].

A widespread mechanism of antimicrobial resistance (AMR) is acquired resistance, when bacteria obtain resistance genes from other bacteria by horizontal gene transfer (HGT). HGT can occur through a few mechanisms, but plasmids play a major role [3]. Plasmids can increase AMR prevalence at multiple scales, from the bacterial level to the pathogen epidemics and worldwide spread of AMR across species. As a result, multi-drug resistant (MDR), extensive-drug resistant (XDR), and pan-drug resistant (PDR) bacteria have been described [4]. Plasmids are important vehicles for the carriage of other mobile genetic elements (MGE); AMR genes are included in the MGEs located in the plasmid’s variable regions, and often, they are clustered in antimicrobial resistance islands [5]. Plasmids have unique properties: extrachromosomal localization, autonomy of replication, the ability to horizontal transfer during conjugation or mobilization, and evolution through gene exchange [6].

Typing of the plasmids and study of their spread and evolution in different bacterial hosts could improve knowledge concerning the epidemiology and the transmission of plasmid-mediated AMR. There are two main principles of plasmid classification based on replicon (encoded plasmid replication) and motility functions (MOB) typing [7,8]. The results of these methods are not always concordant, and not all plasmids are typeable. In addition, a few methods with higher resolution have been developed and used for studying plasmids such as plasmid multi-locus sequence typing (pMLST) and the analysis of core gene single nucleotide polymorphisms (SNPs). However, only whole-genome sequencing (WGS) allows the determination of plasmid relationships most definitely because of plasmids’ ability to gain, lose, and rearrange the genetic regions, meaning that sets of plasmids, even if they are of the same type, may be different and sometimes show hybrid phylogenetic origins [8]. For example, WGS showed the important role of MOBF12A plasmids in the dissemination and persistence of virulence and antibiotic resistance genes in Gram-negative bacteria. Multiple AMR genes, including ESBL or colistin-resistance genes, were localized on a single F-like plasmid or on two compatible MOBF12A plasmids that were simultaneously presented in bacterial cells [9].

The aim of this Special Issue is to provide a collection of articles that highlight the current impact in the research of the plasmids carrying antimicrobial resistance genes in Gram-negative bacteria. As the Guest Editors, we invite researchers in this field to submit their research articles, review articles, and short communications dedicated to the AMR genes and plasmids in Gram-negative bacteria, plasmid typing, HGT from the human microbiome or animal pathogens, etc.
